# Changing the housing environment to reduce obesity in public housing residents: a cluster randomized trial

**DOI:** 10.1186/s12889-018-5777-y

**Published:** 2018-07-16

**Authors:** Deborah J. Bowen, Lisa M. Quintiliani, Sarah Gees Bhosrekar, Rachel Goodman, Eugenia Smith

**Affiliations:** 1 University of Washington, 1959 Pacific Street NE, Box 357120, Seattle, WA 98195 USA; 2 Boston University, Boston, USA; 3 Boston Housing Authority, Boston, USA

## Abstract

**Background:**

Public housing residents face significant social, economic, and physical barriers to the practice of health behaviors for prevention of chronic disease. Research shows that public housing residents are more likely to report higher rates of obesity, current smoking, disability, and insufficient physical activity compared to individuals not living in public housing. Because these behaviors and conditions may be shaped by the built and social environments in which they live, we conducted a study to test an environmental level diet and physical activity intervention targeting obesity among urban public housing developments.

**Methods:**

This study was a cluster randomized controlled trial of public housing developments, the unit of analysis and randomization. A total of 10 public housing developments were recruited and subsequently randomized to either receive the intervention package or to serve as comparison sites. The year-long intervention included components to change the dietary and physical activity-related environments of the developments. Surveys at baseline and one-year follow-up provided data on changes in behaviors and weight from participants in both intervention and control developments.

**Results:**

Intervention participants significantly changed their eating and activity behaviors and body weight from baseline to one-year follow-up (p’s < .05) while comparison participants reported no significant changes in any study variable.

**Conclusions:**

These data provide initial support for the idea that interventions targeting the environment of public housing developments can assist residents to change unhealthy behaviors and can possibly reduce the high levels of chronic disease among public housing residents.

**Electronic supplementary material:**

The online version of this article (10.1186/s12889-018-5777-y) contains supplementary material, which is available to authorized users.

## Background

Persons living in public housing report two to three times the rates of chronic disease and related negative health behaviors compared to nonpublic housing residents [1, 2]. Specifically, obesity (31.0%), current smoking (34.4%), and insufficient physical activity (61.8%) behaviors are all high among public housing residents even when compared to other urban residents [3]. These conditions and behaviors contribute to elevated chronic disease rates in this population [4] and can be targeted for modification through intervention. Therefore, supporting change in these behaviors among this population is an important goal for health promotion efforts among public housing residents.

Given these disparities, the next major question is the choice of intervention strategy or strategies that might be most effective in helping public housing residents to change obesity-related behaviors and reduce chronic disease risk. The approach of multi-level intervention that combine strategies targeted to both individual and environmental levels recognizes the importance of multiple influences on health behaviors. In general, interventions delivered at the individual level, (i.e.*,* those that seek to influence personal factors such as motivation and knowledge and are often delivered one-on-one) produce short-term change, but are not long lasting in the face of an obesogenic environment [5, 6]. Individual interventions are generally resource intensive, often requiring lots of time and relatively high motivation on the part of the participants. Another problem with individual-level interventions that focus on change in individual behaviors, is that they generally elicit low participation rates when offered to the general public [7].

Environmental change might be a reasonable alternative to individual level interventions, in that they take into account social and physical influences on obesity-related behaviors. Investigators have identified multiple areas included below within environments that can serve as intervention targets for change in nonpublic housing settings [8, 9]. Most urban public housing residents live in city and town neighborhoods where availability of healthy resources within a mile of the housing development is ubiquitous. However, we know from our own previous research that public housing residents prefer to interact with people within their housing development and do not seek intervention opportunities in the neighborhood outside of the development, due to hesitation to use mainstream resources to solve problems [10, 11]. Thus it is important to target the housing development environment itself. Several aspects of the built environment of public housing developments might also be barriers to engagement in healthy activities, including perceived lack of access to healthful food outlets, food cost, safe physical activity resources [12], and adequate structural facilities within the development such as stairwells and walking paths. All are possible targets for intervention. The relative contribution of each of these environmental elements to poor food choices and sedentary activities is unknown for public housing residents.

Other studies have recruited public housing residents into health behavior change interventions. The Live Well, Viva Bien trial was a group randomized trial among adults in public housing complexes, during which participants received a 12-month environmental level (i.e.*,* mobile fruit & vegetable markets) and nutrition education intervention to improve fruit and vegetable intake [13]. For smoking, a community health worker led intervention intervened upon public housing residents who smoked to lead them through motivational interviewing-based counseling. At 12-month follow-up, intervention participants (*n* = 121) were more likely than control participants (*n* = 129) to demonstrate 7-day and 30-day point prevalence smoking abstinence (aOR: 2.60 (95% CI: 1.72–3.94); 2.98 (95% CI: 1.56–5.68), respectively) [14]. Our own qualitative pilot work [15, 16] and those of others [17] suggest public housing residents can identify barriers and facilitators to healthy eating and physical activity and ways to begin to address these obstacles. For instance, 27% of residents endorsed obesity as a health issue [10]. While these studies establish the feasibility and efficacy potential of interventions conducted in public housing settings, there continues to be a gap in the literature for programs in public housing developments targeting obesity, that have multiple components, and target the environmental level.

## Methods

### Purpose and design

The design of the study and its intervention have been previously published [18]. Simply, this was a cluster randomized trial, with public housing developments (PHDs) serving as the unit of randomization and analysis. Here we describe the patterns of change in weight, diet behaviors, and physical activity behaviors between baseline and one-year follow-up (12 months after baseline collection) and how these differ between intervention and comparator PHDs.

#### Participants

Boston Housing Authority currently houses more than 21,000 people under the public housing program. Boston Housing Authority manages 63 housing developments. Of the 63 developments, 38 are designated as elderly/disabled developments and 25 are designated as family developments. For this trial, 24 family developments that had 200 or more residents, that were not planning to undergo renovations that would require residents to move, were eligible to participate. Through a process of obtaining approval from development managers and tenant associations [18], we recruited 10 developments (out of 12 approached) to participate in the Healthy Families study; 5 serving as intervention PHDs and 5 serving as control PHDs. PHDs were randomly assigned to either condition, in matched pairs for size of development and existence of health activities in the development. In accordance with our design, all intervention activities were available to all residents in the PHDs randomized to the intervention group. Residents living in PHDs randomized to the control group did not receive any intervention components.

An evaluation cohort was formed in both intervention and control group PHDs that consisted of a group of female residents and their daughters ages 8–15. The evaluation cohort was randomly selected from the overall PHD population in each intervention and control PHD (see Data Collection). Inclusion criteria were: be female; be aged 18–72; live in one of the recruited PHDs and not planning to move for at least 2 years; have responsibility for a girl age 8–15 (also living in the public housing residence), be English or Spanish speaking, and report being willing to make changes to their diet and physical activity habits if desired. Exclusion criteria were: the adult female resident was not able to complete the survey tools or was not interested in participating. The rationale for selecting this population was that female residents represent over 80% of identified heads of households; furthermore we selected daughters because of the development of obesity during this timeframe (8–15 years old). All study materials were available and used in both English and Spanish.

### Data collection

Survey assistants approached randomly selected apartment units within each of the 10 housing developments and requested participation of the adult women in the unit. Using a standardized protocol, survey assistants assessed individual’s interest in participating and eligibility. If interested and eligible and willing to participate, the adult female resident provided their written informed consent to participate. Next, following a similar process, their daughters, girls aged 8–15, gave verbal assent. Initial sample size estimates indicated that a sample of 200 households would provide adequate power for simple main comparisons of body weight. The survey assistant then administered and recorded responses to the baseline survey (see Measures section) for adult participants and measured the women and girl’s height and weight. Project staff returned to re-assess the original evaluation cohort 1 year after baseline measurement to repeat the same data collection measures and procedures as at baseline. Survey assistants were not aware of study arm during data collection.

#### Measures

##### Baseline/follow-up survey

Measurement difficulties exist for both nutrition and physical activity, and so we used the same strategy to select key single or brief items that have been used before in research studies and compared well with longer measures. Following evidenced-based guidelines for health promotion and weight management, we assessed three nutrition behaviors: fruits and vegetable consumption (“*How many servings of fruits and vegetables do you eat each day*?” with 12 responses ranging from 0 to 11 or more, which was prefaced with pictures representing portion sizes); [19] soda consumption (“*How often do you drink soft drinks or soda pop (regular or diet)*?” with 6 responses ranging from never to 2 or more times per day) [20]; mindless eating (*“How often do you eat food (meals or snacks) while doing another activity, for example, watching TV, working at a computer, reading, driving, playing video games?*” with 5 responses ranging from never to always) [20]. We assessed physical activity in the form of walking for leisure, transport, or exercise during the past week with the following question: “*During the last 7 days, on how many days did you walk for at least 10 minutes at a time in your neighborhood?*” with responses ranging from no walking for more than 10 min at a time or the option to fill in number of days per week and number of minutes per day [21]. Number of days/week was multiplied by minutes/day to calculate minutes of walking per week. To assess walking behavior, we then asked: “*On a typical day how many minutes do you walk in your neighborhood?*”; participants filled in the number of minutes per day. Participants also completed standard questions about socio-demographics (age, race/ethnicity, highest level of education completed, self-rated health), nativity, language spoken at home. To assess self-efficacy to eat more healthfully, we asked “*On a scale of 0 to 10, how sure are you that you will eat less sugar and fat during the next year?*” with 11 responses ranging from 0 (not sure) to 10 (very sure) [22] (Additional file 1).

##### Height and weight measurement

The survey assistant measured the height and weight (baseline and follow-up) of both the mother and daughter using a scale. This was used to calculate body mass index as the main outcome of the study (BMI, kg/m^2,^). A systematic protocol was used that consisted of the following steps: Survey assistants asked participants remove their shoes and subtracted 3 pounds from the scale measurement to account for clothing weight. Electronic scales were used (Health o Meter, Model HDM770DQ1–05 E097BN Sunbeam Products operating as Jarden Consumer Solutions Boca Raton Florida). The maximum measurement for this electronic scale was 300 pounds. If a resident weighed over 300 pounds, the survey assistants would not take a weight measurement that day. Rather, they would return to the participant’s home the following day and weight participants using a second scale (Tanita Body Composition Analyzer, Model TBF-300A Tanita Corporation of America Inc., Arlington Heights, Illinois) that could weigh individuals up to 500 pounds. To measure height, survey assistants taped a tape measure to the wall, asked participants to remove their shoes, and stand with their back facing the wall. Survey assistants then measured height in inches and converted it to inches and feet.

#### Intervention

We have previously published the design and conceptual model of our intervention study [16] defined as multilevel: community, organizational, and consumer nutrition and physical activity environments. Residents of intervention PHDs could participate in any of the intervention activities described here. In our first year we focused intervention activities on adult (mothers), with the intention to focus intervention activities for daughters in the second year. However, due to funding cuts, we stopped the project after 1 year.

##### Lay health advisors

Lay health advisors, called Healthy Living Advocates completed a 14-week training in community health outreach, a long-standing program (12+ years) provided by the PHH-PRC [23, 24]. This program was supplemented by study specific training in which, over 3 days, we trained the Healthy Living Advocates in research processes, study specific intervention activities, and weight management information. Topics covered included further training on protecting patient privacy, obesity-related health risks, and ways to encourage weight loss through diet and physical activity behavior change. The Health Living Advocates passed a post-training assessment to demonstrate their knowledge prior to participation in the trial.

##### Health screenings

We offered monthly screenings for blood pressure, smoking, and diabetes risk [25, 26] for residents to learn about their chronic disease risk and receive referrals to a program (such as attending walking groups or cooking demonstrations), to their own health care provider (such as their primary care physician), or given information about how to obtain a provider if they did not have one. Due to the policy landscape at the time of the project, the majority (> 95%) of non-elderly adults in Massachusetts had health insurance, [27] removing this classic barrier to care. The screenings were advertised by the HLAs for 2 weeks before each scheduled screening event. They were 3–4 h in duration and held in a shared space in the development, were advertised for 2 weeks prior. HLAs attended all screenings to assist with linking residents to the primary care system.

##### Access to healthy food

We selected providing access in the form of a van (Fresh Truck) that sold fruits and vegetables to residents of public housing to provide immediate, affordable, and easy access to healthy food choices [28, 29]. The van visited each intervention housing development weekly. HLAs at each development promoted the van’s offerings and attended the Fresh Truck sessions.

##### Walking groups

Environmental-level barriers to physical activity, particularly among racial/ethnic minority low-income women, have been well documented, including issues with neighborhood safety and lack of places to exercise [30–33]. HLAs were trained as weekly walk group leaders and promoted the groups in the developments via flyers and verbal discussion.

##### Cooking demonstrations

Regular cooking demonstrations offered residents the opportunity to not only obtain nutrition education but also to shape social norms around healthful eating and food preparation practices among friends, family, and neighbors [34–36]. All recipes were tailored to meet the needs of the population both culturally and economically. Demonstrations were held approximately every 3 months per development and were led by a Registered Dietitian. Similar to other intervention activities, HLAs promoted the cooking demonstrations beforehand using flyers and word of mouth. HLAs distributed the included recipes, which focused on foods that were available for purchase from the Fresh Truck, were eligible under the Supplemental Nutrition Assistant Program (SNAP), and/or were approved under the Women, Infants, and Children (WIC) approved.

##### Resource maps

Study staff members created printed maps listing local health-related resources (such as local gyms, walking parks, and places to with healthful eating options) and HLAs handed them out to interested participants at health screenings, food van sessions, walking groups, and cooking demonstrations [11].

##### Process data

Data on indicators of intervention implementation/dosage were collected on three forms (1) Group Activity Form, which gathers information on date, type of event, which development it was held at, which HLA led it, number of attendees, number of steps (if walking group), walk route (if walking group), and participant contact information; (2) HLA Weekly Report, which gathers information on all outreach activities conducted by the HLAs in order to encourage residents to attend intervention events; and (3) Participant Evaluation Form, which gathers information on what participants did and did not like about each of the intervention activities.

### Analysis

Data from the Healthy Family study were collected from PHD respondents nested within PHDs; consequently, all analyses were performed using the PHD as the primary unit of randomization. As the first year of the intervention focused on activities targeted at the adult participants, the current analysis focuses on only the adult data. We first examined the data for differences in key baseline demographic characteristics between study arms, and for differences between dropouts at follow-up vs. those participants that provided data at the 12-month survey, to identify imbalances in the study’s sample that could change the interpretation of our data. These analyses were performed using t-tests or chi square tests, as appropriate.

**Fig. 1:**
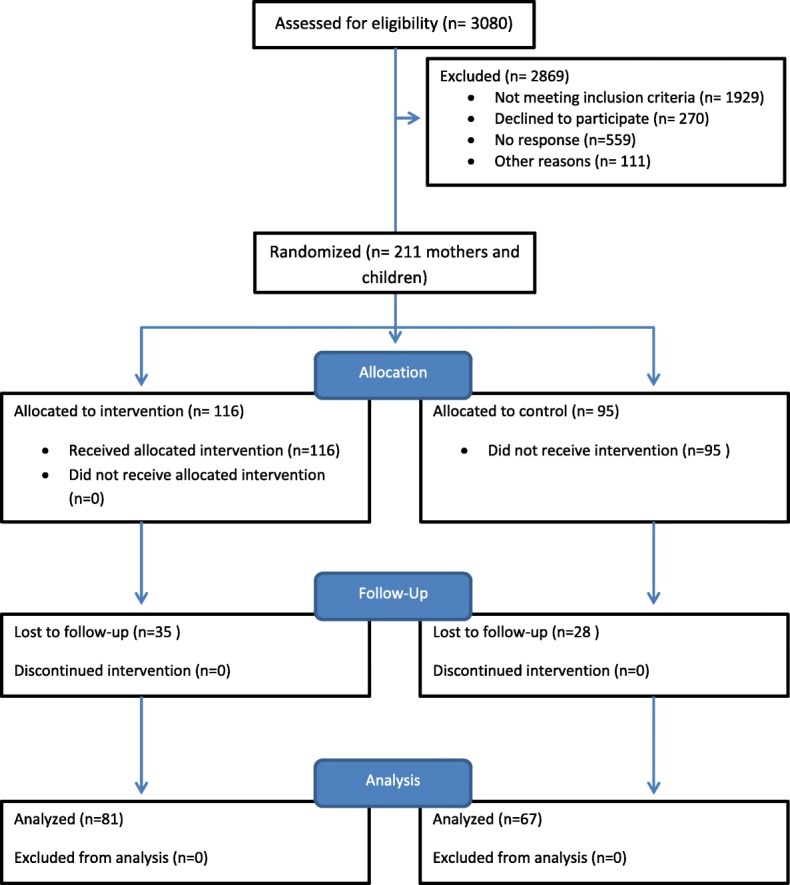
CONSORT document of the Healthy Families study

For the main analysis, we examined differences in the changes from baseline to follow-up in the main outcome data between intervention and comparison PHDs. We also calculated differences at the PHD level between intervention and control, and found no differing patterns from the individual-level data. So we report on individual level data for ease of comparison. Linear mixed models were used to evaluate the study’s intervention, incorporating the design effects of intraclass correlation within PHD and correlation between the two assessment time points (baseline and follow-up). These regression models incorporated a fixed-effect term for treatment condition along with an adjustment for baseline values for the outcome of interest and a PHD was a random effect to account for clustering within development. Models were fit, adjusting for baseline participant level demographic characteristics (age, race, education). There were no baseline patterns of difference in demographic variables, as previously published (18). Data analyses for this paper were generated using SPSS software, version 22. Data were reported using the CONSORT guidelines for reporting [37] (Fig. 1).

## Results

Table 1 presents background variables for the sample of recruited adult participants both overall and by trial arm. Overall, the majority of respondents were Latino (64%), rely on public health insurance (Medicaid or Medicare) (79%), and had a high school education or less (64%). The follow-up rate from baseline to 12-month follow-up was 72%. There were no significant differences in variables in Table 1 between participants who completed only baseline and participants who completed the follow-up.

**Table 1:** Baseline demographic data for participants in the Healthy Families study

	Total^a^ *n* = 211	Control^a^ *n* = 95	Intervention^a^ *n* = 116
Age, years, mean (SD)	38.1 (7.6)	37.1 (6.8)	38.8 (8.2)
Race/Ethnicity^b^			
Asian	3 (1.4)	3 (3.2)	0 (0)
Black or African American	50 (23.8)	26 (27.4)	24 (20.7)
Hispanic/Latino	134 (63.5)	53 (55.7)	81 (69.8)
White	8 (3.8)	5 (5.3)	3 (2.6)
Other	10 (4.7)	4 (4.2)	6 (5.2)
More than one	6 (2.8)	4 (4.2)	2 (1.7)
Language spoken at home			
English	83 (39.3)	40 (42.1)	43 (37.1)
Spanish	104 (49.3)	38 (40)	66 (56.9)
Other	24 (11.4)	17 (17.9)	7 (6)
Born in the U.S.			
Yes	72 (34.1)	29 (30.5)	43 (37.1)
No	139 (65.9)	66 (69.5)	73 (62.9)
Highest level of education			
< High school	60 (28.4)	21 (22.1)	39 (33.6)
High school graduate/GED	75 (35.6)	39 (41.1)	36 (31.1)
Some college or technical college	48 (22.7)	24 (25.3)	24 (20.7)
College graduate	25 (11.9)	10 (10.5)	15 (12.9)
Other	3 (1.4)	1 (1)	2 (1.7)
Health insurance^b^			
Private insurance	16 (7.6)	4 (4.2)	12 (10.4)
Medicaid, MassHealth or Commonwealth Care	164 (77.7)	78 (82.1)	86 (74.1)
Medicare	3 (1.4)	1 (1)	2 (1.7)
Free care	10 (4.7)	4 (4.2)	6 (5.2)
Other	5 (2.4)	5 (5.3)	0 (0)
None	1 (0.5)	0 (0)	1 (0.8)
More than one	12 (5.7)	3 (3.2)	9 (7.8)
Self-rated health			
Excellent	28 (13.3)	13 (13.7)	15 (12.9)
Very good	31 (14.7)	15 (15.8)	16 (13.8)
Good	86 (40.7)	38 (40)	48 (41.4)
Fair	58 (27.5)	26 (27.4)	32 (27.6)
Poor	8 (3.8)	3 (3.1)	5 (4.3)

^a^Numbers represent n

^b^Subjects were able to choose multiple answers

### Process data

HLAs were asked to complete forms for specific intervention activities, documenting participation by residents. In total, over 1 year, HLAs documented 12 health screenings (128 total participants, 68 who live in the development), 20 walking groups (62 total participants, 27 who live in the development), and 8 cooking demonstrations (45 total participants, 36 who live in the development). Participants attending activities were also asked to complete a form documenting their perceptions of the activity. For health screenings, 6 evaluation forms from participants were returned; however 51 evaluation forms were returned for walking groups and 37 evaluation forms for cooking demonstrations. Table 2 presents process data for these specific intervention components. As seen in this table, participants who responded considered the intervention useful and the activities achievable. Specifically, both the existence of the activity and the social contact of the activity were seen as helpful by most respondents.

**Table 2:** Perceptions of residents attending intervention activities: walking groups and cooking demonstrations

	Walking groups (*n* = 51 forms)	Cooking demonstrations (*n* = 37 forms)
	%	%
Difficulty of activity		
Very difficult/difficult	22	3
Neutral	37	3
Easy/very easy	39	92
blank	2	3
Total time spent on activity		
Very difficult/difficult	20	3
Neutral	39	11
Easy/very easy	39	84
blank	2	3
Usefulness of HLA during activity		
Not at all/slightly useful	0	3
Somewhat useful	4	5
Very/extremely useful	96	84
blank	0	8
Usefulness of fellow activity participants		
Not at all/slightly useful	4	3
Somewhat useful	6	0
Very/extremely useful	90	89
Blank	0	8
Physical benefits from participating in activity		
Not at all/slightly useful	2	5
Somewhat useful	0	13
Very/extremely useful	98	76
Blank	0	5
Social benefits from participating in activity		
Not at all/slightly useful	2	3
Somewhat useful	0	0
Very/extremely useful	96	95
Blank	2	3
Activity makes me want to try new things		
Strongly disagree/disagree	0	3
Neutral	0	5
Agree/strongly agree	100	84
Blank	0	8
Activity will help me control my weight		
Strongly disagree/disagree	0	3
Neutral	2	3
Agree/strongly agree	94	92
Blank	4	3

### Study outcome data

**Fig. 2:**
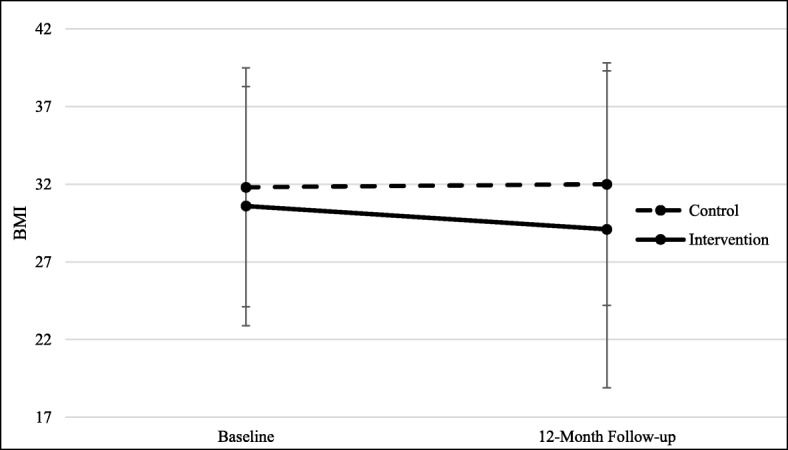
Body mass Index (BMI) at baseline and 12-month follow-up for participants in intervention and control public housing developments in the Healthy Families study

The changes in body weight, calculated as Body Mass Index or BMI, for intervention and control participants in the Healthy Families study significantly changed in intervention compared to control groups (Fig. 2). We observed a small but significant difference between intervention and control participants from baseline to follow-up. Intervention participants on average reduced their BMI by 1.5 points (from 30.6 (sd = 7.7) to 29.1 (sd = 10.2)) while comparison participants increased their BMIs by 0.2 BMI points on average (from 31.8 (sd = 7.7) to 32.0 (sd = 7.8)). This difference was significant, in both the unadjusted (*p* < 0.05) and adjusted (*p* = 0.04) analyses.

The changes from baseline to follow-up in measured dietary and activity behaviors are presented in Table 3. There were no significant differences between intervention and control participants in any of the baseline values for dietary and activity behaviors. Across the 5 behaviors studied, there were very minimal changes reported by the control participants from baseline to 12-month follow-up, as can be seen in Table 3. In contrast, in four of the five behaviors measured, there were significant improvements from before to after intervention exposure in the intervention participants. Statistically significant change score differences were seen in mean fruit and vegetable intake, in % of individuals reporting each fast food less than once per week, in the % of participants reported as inactive, and in minutes of walking in the neighborhood per day (all *p* values < 0.05 with paired t-tests or chi square tests as appropriate). Adjustment for baseline value of the outcome variable and for housing development did not alter this pattern of significance in the main outcomes analyzed using multiple regression. The effects of intervention versus control condition was significant in linear regression for both fruit and vegetable consumption (*p* = 0.03) and for minutes per day of walking (*p* = 0.007).

**Table 3:** Pre- and post-intervention values in obesity-related behaviors among participants in the Healthy Families study

Variable	Baseline		Follow-up		Change score		Beta or odds ratio/Adjusted p value^a^/CI
	C	I	C	I	C	I	
	n = 95	n = 116	*n* = 67	*n* = 81			
Fruits & vegetables, mean per/day (sd)	1.9 (2.0)	2.0 (2.1)	2.0 (2.0)	3.6 (2.5)	+.1	+ 1.6	2.30/.03
Soft drinks, % once per week or more	30%	31%	29%	25%	-1	-6	–
Fast food, % < once per week	66%	70%	65%	55%	-1	−15	1.7/.04/1.12–3.6
% Inactive	82%	89%	80%	59%	−2	−30	2.4/.007/1.8–5.6
Walking in neighborhood, min/day	19.6 (24.6)	19.8 (39.8)	19.5 (32.2)	30.5 (42.7)	−.1	+ 10.7	2.7/.01

^a^Adjusted for age, race, education level, baseline value of variable

## Discussion

The purpose of this study was to test the effects of an environmental-level package of interventions on public housing residents’ obesity and obesity-related behaviors. We aimed to evaluate the effectiveness of a package of intervention opportunities, each element targeting a different aspect of the food and activity environment, on reducing body weight and increasing healthy eating and healthy activity behaviors of public housing residents. We had collected pilot data previously that identified the main barriers and issues that interfered with pursuit of health in these residents. With the guidance of our participating colleagues, we designed an environmentally focused intervention targeting the key behaviors related to obesity. The intervention was successful in reducing weight levels, and in increasing healthy eating (in the form of fruit and vegetable intake and fast food consumption) and healthy physical activity (in the form of walking) over a 12-month period. Four of the five behavioral outcomes were in the hypothesized direction and were consistent in magnitude of change with each other.

From these data we do not have specific explanations as to why this intervention was efficacious. Trials of this design and type are not meant to fully understand which components were the most powerful or useful in producing these changes in the residents. Increased recent attention to trial designs that allow researchers to identify the specific and most active ingredients of change [38] could be applied to the next round of research in this area. This knowledge would help future interventions to be more efficient and potentially increased in potency. Testing multiple components in a fully or partially factorial design would address the issue and would provide much needed knowledge of specific active ingredients.

We ended this study after only 1 year of active intervention and follow-up due to reductions in funding. We originally intended to continue for at least 3 years to enable us to better understand and support sustainability of this type of intervention. We designed the components to be sustainable, together with community partners, but we have no data on the actual events that took place after funding ended. The long-term changes are important to measure and explore, as chronic disease prevention requires changes to occur over years instead of months [39]. Certainly, several of the partners intended to assist with continuing long-term support of healthy behaviors among public housing residents but we do not have data as to their success.

We decided to limit our findings to those data collected from adult participants, and not the daughters, given that the intervention emphasis on daughters was to have happened in the second and third years. Changes in children would be a possible area for future research to pursue. Findings from a survey conducted among public housing communities in Maryland found that the social environment of carers was related to health behaviors among children [40] Future work should aim to more fully incorporate the family unit into intervention delivery activities conducted in public housing.

There were several design choices that limited the generalizability of the study findings. First, the measures of healthy eating and physical activity were relatively minimal. Dietary intake is difficult and time consuming to measure, and so in the interest of minimizing participant burden and increasing total survey completion, we decided to measure key single behaviors related to obesity for which there were existing measures. Measurement of these behaviors is in general complex; there are no brief but perfect measures to use in community-based studies. Still, in future research we must increase attention toward measuring outcomes in a more thorough manner using multiple measures of different inherent biases and shortcomings. We were only able to measure outcomes at baseline and one follow-up, and this limits our understanding of the patterns of change reported by participants. We did not include any objective or observational measures, such as electronic activity monitoring or biomarkers of eating. There are few such biomarkers that track well onto healthy eating, but this is an area for future research as well. We also did not measure any variables that could provide insight into the mechanism of successful changes, although literature on environmental level literature suggests the following variables may have been responsible for these changes. This is a considerable gap, as we now have little idea of why the intervention worked. This too can be rectified in future research projects in public housing.

## Additional file

Additional file 1: PHD survey baseline. This survey was used to collect baseline data for the present study. (DOCX 305 kb)

## Abbreviations

BHA: Boston Housing Authority
BMI: Body Mass Index
HLA: Healthy Living Advocates
PHD: Public Housing Development
PHH-PRC: Partners in Health and Housing—Prevention Research Center
