# Supplementary material for: Changing the housing environment to reduce obesity in public housing residents: a cluster randomized trial
Source: BMC Public Health. 2018 Jul 16;18:883. doi: 10.1186/s12889-018-5777-y (PMC6048807; doi:10.1186/s12889-018-5777-y)
Supplement: Supplementary file 1 — PHD survey baseline. This survey was used to collect baseline data for the present study. (DOCX 305 kb) [file 12889_2018_5777_MOESM1_ESM.docx]

Place ID label here

**PHH-PRC**

**Healthy Families Survey**

**Baseline Form**

**Date: __ __ / __ __ / __ __ Form:1**

**Screening Staff ID: ___ ___**

**Development___________**

***Part 1: Basic Information***

**I am going to start by asking you a few basic questions about yourself, if you need to stop at any point let me know and we can take a break and start again when you are ready, okay?**

**1) What race or races do you most closely identify with? *Read Options—Check all that apply***

- **Asian^1^**
- **American Indian or Alaska Native^1^**
- **Black or African American^1^**
- **Hispanic/Latino^1^**
- **Native Hawaiian or other Pacific Islander^1^**
- **White^1^**
- **Other^1^: ___________________________**

**2) What language do you speak at home with your family most often? *Check only one***

- **English^1^**
- **Spanish^2^**
- **Chinese^3^**
- **Vietnamese^4^**
- **Portuguese^5^**
- **Portuguese Creole/Cape Verdean Creole^6^**
  - **Haitian Creole^7^**
  - **Arabic^8^**
  - **French^9^**
  - **Amharic^10^**
  - **Other^11^: __________________**

**3) Were you born in the United States?**

**🞎 Yes^1^ 🞎 No^2^**

**4) What is the highest grade of school you completed?**

- **Never attended school or only attended kindergarten^1^**
- **Elementary school (Grades 1 through 8)^2^**
- **Some high school^3^**
- **High school graduate^4^**
- **Some college or technical school^5^**
- **College graduate^6^**

**Other education (please specify)^7^: __________________________**

**5) Do you have health insurance?**

***IF YES—*What kind?**

***IF NECESSARY—* *Go through options with participant. Check all that apply***

- **Private Insurance (for example: Blue Cross, Harvard Pilgrim)**
  - **Medicaid, MassHealth, or Commonwealth Care^1^**
  - **Medicare**
  - **Free Care^1^**
  - **Other^1^:___________________**
  - **None^1^**
  - **Don’t know^1^**

1. **Who lives with you? Read Options—Check all that apply**

- **I live alone**
- **I live with my husband or partner**
- **I live with my children**
- **I live with my brother and/or sister**
- **I live with other relatives**
- **I live with friends**
- **Other (please describe**

1. **How many people including yourself live in your household as members of your family (whom you support or who contribute to supporting your family? Read options—Mark one answer**

| **None**  **_1_** | **One**  **_1_** | **Two**  **_1_** | **Three**  **_1_** | **Four**  **_1_** | **Five or more**  **_1_** |
| --- | --- | --- | --- | --- | --- |

**How many of these people are under 18 years old? Read options—Mark one answer**

| **None**  **_1_** | **One**  **_1_** | **Two**  **_1_** | **Three**  **_1_** | **Four**  **_1_** | **Five or more**  **_1_** |
| --- | --- | --- | --- | --- | --- |

**How many are between 18 and 64 years old (including yourself)? Mark one answer**

| **None**  **_1_** | **One**  **_1_** | **Two**  **_1_** | **Three**  **_1_** | **Four**  **_1_** | **Five or more**  **_1_** |
| --- | --- | --- | --- | --- | --- |

**How many are 65 years or older? Mark one answer**

| **None**  **_1_** | **One**  **_1_** | **Two**  **_1_** | **Three**  **_1_** | **Four**  **_1_** | **Five or more**  **_1_** |
| --- | --- | --- | --- | --- | --- |

1. **What is your current marital status? Read Options—Mark one answer**

- **Never married**
- **Divorced or separated**
- **Widowed**
- **Presently married**
- **Living in a marriage-like relationship**

**If not married or not living in a marriage-like relationship skip to 12**

1. **Which category below best describes the highest level of school your husband or partner has completed? Read options—Mark one answer**

- **Didn’t go to school**
- **Grade school (1-4 years)**
- **Grade school (5-8 years)**
- **Some high school (9-11 years)**
- **High school diploma or G.E.D.**
- **Vocational or training school AFTER highschool graduation**
- **Some college or associates degree**
- **College graduate or Baccalureate degree**
- **College or professional school AFTER college graduation**
- **Masters degree**
- **Doctoral Degree (Ph.D, M.D. etc)**

1. **What is your husband or partner’s current job status?**

- **Not working**
- **Retired**
- **Homemaker, raising children, care of others**
- **Employed (full time or part time)**
- **Disables, unable to work**
- **Other (specify) __________**

1. **Which statement below best describes your husband or partner’s job? If not working now, which best describes your partner’s last job? Read options—Mark one answer**

- **HOMEMAKER, RAISING CHILDREN, CARE OF OTHERS**
- **MACHINE OPERATOR** For example: meter reader, installer, truck driver, etc
- **MANUAL LABOR** For example: maintenance worker, construction work, warehouse

worker, utility worker, material handler, etc.

- **SKILL** OR **CRAFT** For example: building trades, hourly paid supervisor, line worker,

technician, mechanic, repairer, etc.

- **SCIENTIFIC TECHNICAL WORK** For example: computer programmer, dental assistant, vocational/practical nurse, computer operator, engineering aide, lab technician, etc.
- **SERVICE WORK** For example: firefighter, security worker, custodian/maid, cook, waitress/waiter, seamstress/tailor, farmer, forestry or fishing occupations, cafeteria worker, store clerk, gas attention attendant, etc.
- **CLERICAL, OFFICE** OR **SALES WORK** For example: billing, customer service, receptionist, cashier, telephone operator, administrative support, typist, travel agent, bank teller, etc.
- **PROFESSIONAL, MANAGERIAL** OR **ADMINISTRATIVE WORK** For example: engineer, teacher, registered nurse, lawyer, accountant, salaried manager, personnel worker, etc.
- **OTHER (SPECIFY)** __________________________________________

1. **During the past two weeks how often have you been bothered by having little interest or pleasure in doing things? *Read Options***

- **Not at all**
- **Several days**
- **More than half the days**
- **Nearly every day**
- **Not sure (Do not read aloud. Voluntary only – included given that this survey will be administered by student/RHA team)**
- **Decline to answer (Voluntary only)**

1. **During the past two weeks how often have you been bothered by feeling down, depressed or hopeless? *Read Options***

- **Not at all**
- **Several days**
- **More than half the days**
- **Nearly every day**
- **Not sure (Do not read aloud. Voluntary only – included given that that this survey will be administered by student/RHA team)**
- **Decline to answer (Voluntary only)**

| During the Past week… | Rarely or none of the time (less than 1 day) | Some or a little of the time (1-2 days) | Occasionally or a moderate amount of the time (3-4 days) | All of the time (5-7 days) |
| --- | --- | --- | --- | --- |
| I was bothered by things that usually don’t bother me read options | **_0_** | **_1_** | **_2_** | **_3_** |
| I had trouble keeping my mind on what I was doing read options | **_0_** | **_1_** | **_2_** | **_3_** |
| I felt depressed read options | **_0_** | **_1_** | **_2_** | **_3_** |
| I felt that everything I did was an effort | **_0_** | **_1_** | **_2_** | **_3_** |
| I felt hopeful about the future | **_0_** | **_1_** | **_2_** | **_3_** |
| I felt fearful | **_0_** | **_1_** | **_2_** | **_3_** |
| My sleep was restless | **_0_** | **_1_** | **_2_** | **_3_** |
| I was happy | **_0_** | **_1_** | **_2_** | **_3_** |
| I felt lonely | **_0_** | **_1_** | **_2_** | **_3_** |
| I could not “get going” | **_0_** | **_1_** | **_2_** | **_3_** |

**Okay, now I am going to ask you some questions about your internet use.**

1. **Do you ever use the Internet to send or read email?**

- **yes**
- **No**

***IF YES—***

1. **Did you send or receive emails yesterday?**

- **yes**
- **No**

1. **How often do you use the internet or send emails while you are at your house? *Read Options***

- **Several times a day**
- **About once a day**
- **3-5 days a week**
- **1-2 days a week**
- **Every few weeks**
- **Less often or never**

1. **How often do you use the internet or send emails from your job? *Read Options Again***

- **Several times a day**
- **About once a day**
- **3-5 days a week**
- **1-2 days a week**
- **Every few weeks**
- **Less often or never**

1. **How often do you use the internet to send emails from someplace other than your house or work, for example: from the library, or even somebody else’s home? *Read Options Again***

- **About once a day**
- **3-5 days a week**
- **1-2 days a week**
- **Every few weeks**
- **Less often or never**

1. **Do you ever use your phone or other portable devices such as a Blackberry Ipad or other tablets to use the internet?**

- **yes**
- **No**

***IF YES—***

1. **Did you use your phone or other portable device to access the internet yesterday?**

- **yes**
- **No**

1. **Each day, how many text messages do you think that you send or recieve? *Read Options***

- **0**
- **1 to 10**
- **11 to 20**
- **21 to 50**
- **51 to 100**
- **101 to 200**
- **More than 200**

***This question is very iffy to me considering the participants. Could cause confusion***

1. **At your house, how are you connected to the internet? *Walk Through Options***

- **a dial-up telephone line**
- **a DSL-enabled phone line**
- **a cable modem**
- **a satellite connection**
- **a fixed wireless provider, such as Verizon or Time Warner**
- **other wireless connection, such as an AirCard or cell phone**
- **a fiber optic connection, such as FIOS**
- **a T-1 connection**
- **other**
- **don't know**
- **does not apply**

1. **Do you ever use the internet to look for health, diet, or fitness information?**

- **yes**
- **No**

1. **Do you ever use the internet for websites like Facebook, Twitter, Myspace or LinkedIn?**

- **yes**
- **no**

***IF YES—***

1. **Did you use the internet for one of these websites yesterday?**

- **yes**
- **no**

1. **Do you have a FaceBook account?**

- **yes**
- **no**

1. **Do you have a Twitter account?**

- **yes**
- **no**

1. **Do you have a MySpace account?**

- **yes**
- **no**

**In the next section of the survey I am going to ask you questions about your physical activity.**

|  | **In a 7 day period, during your free time, how often do you do an activity or exercise long enough to make you sweat? *Read Options*** | | |
| --- | --- | --- | --- |
|  | **^1^ Often** | **^2^ Sometimes** | **^3^ Never/Rarely** |
|  | | | |

**Now I would like to ask you some questions about your health.**

| 1. **How often do you eat breakfast, lunch, or dinner at a fast food restaurant such as Mcdonalds, Burgerking, Wendy’s, Arby’s, Pizza Hut, or KFC?** | | |
| --- | --- | --- |
| **_____ times per week OR** | **______ times per month OR** | **_____ times per year** |

|  | **In general, would you say your health is? *Read Options—Mark One Answer.*** | | | | | |
| --- | --- | --- | --- | --- | --- | --- |
|  | **_1_**  **Excellent** | **_2_**  **Very good** | **_3_**  **Good** | **_4_**  **Fair** | **_5_**  **Poor** |  |
|  |  |  |  |  |  |  |

| 1. **The following questions are about activities you might do during a typical day. Does your health now limit you in these activities? If so, how much?** | **YES,**  **LIMITED**  **A LOT** | **YES,**  **LIMITED**  **A LITTLE** | **NO,**  **NOT LIMITED**  **AT ALL** |
| --- | --- | --- | --- |
| **a. Moderate activities, such as moving a table, pushing a vacuum cleaner, bowling, or playing golf? Read Options** | **1** | **2** | **3** |
| **b. Climbing several flights of stairs? Read Options** | **1** | **2** | **3** |

|  | | | | | |
| --- | --- | --- | --- | --- | --- |
| 1. **During the past 4 weeks, have you had any of the following problems with your work or other regular daily activities as a result of your physical health?** | **NO,**  **NONE**  **OF THE TIME** | **YES,**  **A LITTLE**  **OF THE TIME** | **YES,**  **SOME**  **OF THE TIME** | **YES,**  **MOST**  **OF THE TIME** | **YES,**  **ALL**  **OF THE TIME** |
| **a. Accomplished less than you would like. Read Options** | **1** | **2** | **3** | **4** | **5** |
| **b. Were limited in the kind of work or other activities. Read Options** | **1** | **2** | **3** | **4** | **5** |

|  | | | | | |
| --- | --- | --- | --- | --- | --- |
| 1. **During the past 4 weeks, have you had any of the following problems with your work or other regular daily activities as a result of any emotional problems (such as feeling depressed or anxious)?** | **NO,**  **NONE**  **OF THE TIME** | **YES,**  **A LITTLE**  **OF THE TIME** | **YES,**  **SOME**  **OF THE TIME** | **YES,**  **MOST**  **OF THE TIME** | **YES,**  **ALL**  **OF THE TIME** |
| **a. Accomplished less than you would like. Read Options** | **1** | **2** | **3** | **4** | **5** |
| **b. Didn't do work or other activities as carefully as usual. Read Options** | **1** | **2** | **3** | **4** | **5** |

1. **During the past 4 weeks, how much did pain interfere with your normal work (including both work outside the home and house work)? Read Options**

| **NOT AT ALL** | **A LITTLE BIT** | **MODERATELY** | **QUITE A BIT** | **EXTREMELY** |
| --- | --- | --- | --- | --- |
| **1** | **2** | **3** | **4** | **5** |

**These questions are about how you feel and how things have been with you during the past 4 weeks. For each question, please give the one answer that comes closest to the way you have been feeling.**

| 1. **How much of the time during the past 4 weeks:** | | | | | | |
| --- | --- | --- | --- | --- | --- | --- |
|  | **ALL OF**  **THE TIME** | **MOST OF**  **THE TIME** | **A GOOD BIT OF**  **THE TIME** | **SOME OF**  **THE TIME** | **A LITTLE OF**  **THE TIME** | **NONE OF**  **THE TIME** |
| **a. Have you felt calm and peaceful? Read Options** | **1** | **2** | **3** | **4** | **5** | **6** |
| **b. Did you have a lot of energy? Read Options** | **1** | **2** | **3** | **4** | **5** | **6** |
| **c. Have you felt downhearted and blue? Read Options** | **1** | **2** | **3** | **4** | **5** | **6** |

1. **During the past 4 weeks, how much of the time has your physical health or emotional problems interfered with your social activities (like visiting with friends, relatives, etc.)? Read Options**

| **ALL OF THE TIME** | **MOST OF THE TIME** | **SOME OF THE TIME** | **A LITTLE OF THE TIME** | **NONE OF THE TIME** |
| --- | --- | --- | --- | --- |
| **1** | **2** | **3** | **4** | **5** |

1. **Compared to one year ago, how would you rate your physical health in general now? Read Options**

| **MUCH BETTER** | **SLIGHTLY BETTER** | **ABOUT THE SAME** | **SLIGHTLY WORSE** | **MUCH WORSE** |
| --- | --- | --- | --- | --- |
| **1** | **2** | **3** | **4** | **5** |

1. **Compared to one year ago, how would you rate your emotional problems (such as feeling anxious, depressed or irritable) now? Read Options**

| **MUCH BETTER** | **SLIGHTLY BETTER** | **ABOUT THE SAME** | **SLIGHTLY WORSE** | **MUCH WORSE** |
| --- | --- | --- | --- | --- |
| **1** | **2** | **3** | **4** | **5** |

***Show Participants This Chart—Walk Through The Serving Sizes for Each. Ensure understanding before moving on Maybe laminate a colored/enlarged/simplified copy and keep that separate to bring out and walk the participants through***

| **Sample Serving Size** | | | | |
| --- | --- | --- | --- | --- |
| **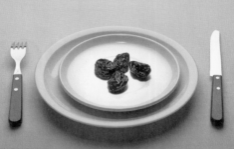**  **A serving is ¼ cup  dried fruit** | **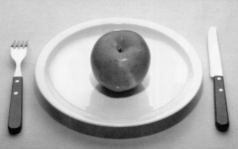**  **A serving is one medium piece of fruit** | **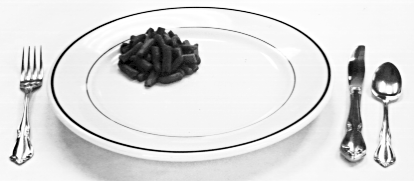**  **A serving is ½ cup of cooked vegetables** | **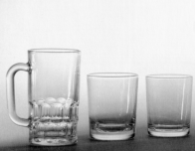**  **A serving is 6 ounces of 100% fruit juice** | **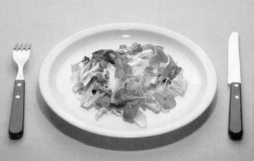**  **A serving is  1 cup of salad** |
| **Note: • Please include vegetables in soups, stir-fry, and other mixed dishes.**  **• “100% juice from concentrate” is considered juice.**  **• Other fruit drinks such as “fruit juice cocktail,” “juice beverage,” or “10%” juice   are not counted as fruit juice.** | | | | |

| 1. **.** | | **About how many servings of fruits and vegetables do you eat each day? *Read Options—Mark one answer.*** | |
| --- | --- | --- | --- |
|  | \| **0** \| **1** \| **2** \| **3** \| **4** \| **5** \| **6** \| **7** \| **8** \| **9** \| **10** \| **11 or more** \| \| --- \| --- \| --- \| --- \| --- \| --- \| --- \| --- \| --- \| --- \| --- \| --- \| | |  |

| 1. **How often do you drink soft drinks or soda pop (*regular or diet*)?**   ***Read Options—Mark one answer*** |  | 1. **How often do you eat food (meals or snacks) while doing another activity, for example, watching TV, working at a computer, reading, driving, playing video games? *Read Options—* *Mark one answer*** |
| --- | --- | --- |
| \| **^1^ Never** \| \| --- \| \| **^2^ Less than once a week** \| \| **^3^ About once a week** \| \| **^4^ 2-5 times per week** \| \| **^5^ About once a day** \| \| **^6^ 2 or more times per day** \| |  | \| **^1^ Never** \| \| --- \| \| **^2^ Seldom** \| \| **^3^ Sometimes** \| \| **^4^ Most of the time** \| \| **^5^ Always** \| |

**I am now going to ask you some questions about the community that you live in.**

|  | **How many years have you lived in your community? *Mark one answer*** | | | | | | |  |
| --- | --- | --- | --- | --- | --- | --- | --- | --- |
|  | **^1^** | **Less than one year** | | |  |  |  |  |
|  | **^2^** | **One to five years** | | |  |  |  |  |
|  | **^3^** | **Six to ten years** | | |  |  |  |  |
|  | **^4^** | **Eleven to twenty years** | | |  |  |  |  |
|  | **^5^** | **More than twenty years** | | |  |  |  |  |
|  | **^6^** | **All my life** | | |  |  |  |  |
|  | **^7^** | **Don’t know** | | |  |  |  |  |
|  |  | | | | | | | |
|  | **Do you expect to be living in your community five years from now? Mark one Answer** | | | | | | | |
|  | **^0^ No** | | **^1^ Yes** | **^3^ Don’t Know** | |  |  |  |

|  | 1. **Overall, how would you rate your community as a place to live? Read Choices—Mark one answer** | | | |
| --- | --- | --- | --- | --- |
|  | **^1^** | **Excellent** |  |  |
|  | **^2^** | **Good** |  |  |
|  | **^3^** | **Only fair** |  |  |
|  | **^4^** | **Poor** |  |  |

| **These questions are about what is available in your neighborhood.** | | | | | |
| --- | --- | --- | --- | --- | --- |
|  | ***Give your best guess***  ***Mark one answer*** | **Strongly agree** | **Agree** | **Disagree** | **Strongly Disagree** |
|  | **There are many places to go for example: stores, businesses, churches, parks) within easy walking distance which means within one mile of my home. *Read Options*** | **^1^** | **^2^** | **^3^** | **^4^** |
|  | **There are sidewalks on most of the streets in my neighborhood. *Read Options*** | **^1^** | **^2^** | **^3^** | **^4^** |
|  | **There is a high crime rate in my neighborhood. *Read Options*** | **^1^** | **^2^** | **^3^** | **^4^** |
|  | **The sidewalks in my neighborhood are well maintained meaning that they are paved, even, and do not have a lot of cracks** | **^1^** | **^2^** | **^3^** | **^4^** |
|  | **The majority of food available in my neighborhood is fast-food.** | **^1^** | **^2^** | **^3^** | **^4^** |
|  | **There are many grocery stores and supermarkets in my neighborhood.** | **^1^** | **^2^** | **^3^** | **^4^** |
|  | **There are many places to exercise in my neighborhood like gyms or parks** | **^1^** | **^2^** | **^3^** | **^4^** |

| **Give your best guess mark one answer** | **Not a problem** | **Some Problem** | **Serious problem** |
| --- | --- | --- | --- |
| 1. **Litter in the streets**   **Read options** | **^1^** | **^2^** | **^3^** |
| 1. **Smells and fumes**   **Read options** | **^1^** | **^2^** | **^3^** |
| 1. **Walking around after dark**   **Read options** | **^1^** | **^2^** | **^3^** |
| 1. **Problems with dogs** | **^1^** | **^2^** | **^3^** |
| 1. **Noise from traffic and other homes** | **^1^** | **^2^** | **^3^** |
| 1. **Lack of entertainment (restaraunts, theatres, parks etc)** | **^1^** | **^2^** | **^3^** |
| 1. **Traffic and road safety** | **^1^** | **^2^** | **^3^** |
| 1. **Places to shop** | **^1^** | **^2^** | **^3^** |
| 1. **Vandalism** | **^1^** | **^2^** | **^3^** |
| 1. **Disturbances by neighbors or young people** | **^1^** | **^2^** | **^3^** |

| 1. **Think about the time you spend walking in your neighborhood. This includes walking for any purpose, such as for fun, exercise, or walking from place to place. This question may need some work just for understanding sake, the wording is a little bit complicated, need to think through how to simplify this.** |
| --- |
| **During the *last 7 days,* on how many days did you walk for at least 10 minutes at a time in your neighborhood and how many minutes would you say that you walked each day? Give your best guess.** |
| **No walking for more than 10 minutes at a time in my neighborhood. *Go to question 121***  **_________ days per week and ______ _______ minutes per day**    ***(example* 3 days per week and 2 0 minutes per day*)*** |
| **b) On a *normal day* how many minutes do you walk in your neighborhood? *Give your best guess*** |
| **___ ___ minutes per day** |

**Think about the time you spend sitting while at work, at home, while doing course work and during leisure time. This may include time spent sitting at a desk, visiting friends, reading or sitting or lying down to watch television.**

1. **During the last 7 days, how much time did you usually spend sitting on a weekday *(for***

***example Monday or Tuesday)? (Give your best guess)***

____ ____: ____ ____ per day

hours minutes

1. **During the last 7 days, how much time did you usually spend sitting on a weekend day**

***(on Saturday or Sunday)? (Give your best guess)***

____ ____: ____ ____ per day

hours minutes

|  | Give your best guess | No, not in past 4 weeks | Yes, less than once a week | Yes, 1 or 2 times a week | Yes, 3 or 4 times a week | Yes, 5 or more times a week |
| --- | --- | --- | --- | --- | --- | --- |
| a. | **Did you have trouble falling asleep? Read Options** | **^0^** | **^1^** | **^2^** | **^3^** | **^4^** |
| b. | **Did you wake up several times during the night? Read Options** | **^0^** | **^1^** | **^2^** | **^3^** | **^4^** |
| c. | **Did you wake up earlier than you had planned to? Read Options** | **^0^** | **^1^** | **^2^** | **^3^** | **^4^** |
| d. | **Did you have trouble getting back to sleep after you woke up too early? Read Options** | **^0^** | **^1^** | **^2^** | **^3^** | **^4^** |

|  | **Give your best guess** | **Very sound or restful** | **Sound or restful** | **Average Quality** | **Restless** | **Very restless** |
| --- | --- | --- | --- | --- | --- | --- |
|  | **Overall, how was your typical night’s sleep during the past 4 weeks? Read Options** | **^0^** | **^1^** | **^2^** | **^3^** | **^4^** |

**Have you ever been told you had a health problem?**

- **Yes^1^ *^If yes—what?^*^___________________________________________________^**
- **No^2^**

1. **Now I’d like to ask you a few questions that relate to your experience with alcohol, cigarettes, and other drugs.**

**In the past year, how often have you taken and used the following?**

|  | **Never** | **Once or twice** | **Monthly** | **Weekly** | **Daily or Almost daily** |
| --- | --- | --- | --- | --- | --- |
| **Alcohol *Read Options*** | **^0^** | **^1^** | **^2^** | **^3^** | **^4^** |
| **Tobacco *Read Options*** | **^0^** | **^1^** | **^2^** | **^3^** | **^4^** |
| **Prescription Drugs for Non-medical reasons** | **^0^** | **^1^** | **^2^** | **^3^** | **^4^** |
| **Other drugs** | **^0^** | **^1^** | **^2^** | **^3^** | **^4^** |

**
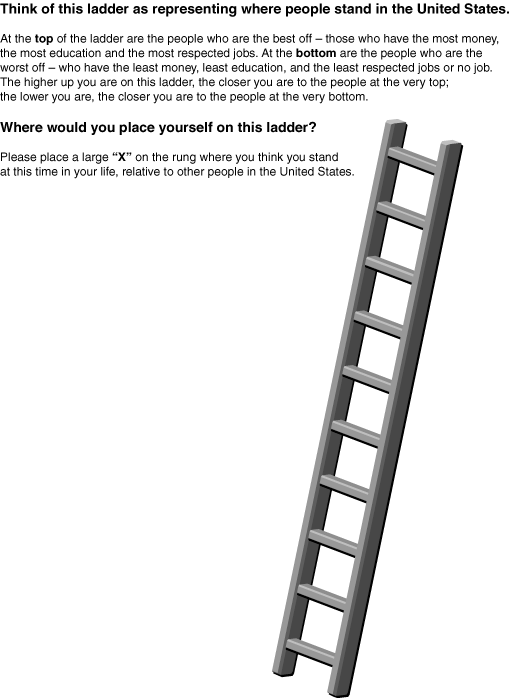
**

Along with other diagram thinking that maybe having a laminated and enlarged copy of this image to aid in the explanation for the participant would be very useful.

***Present Ladder—Read Instructions. Ensure Understanding before asking them to point to where they think they belong. Mark answer and confirm their answer.***

**Thank you so much for your time completing the survey**

**SCREENING STAFF ONLY**

1. **Adult Height: ____ ft ______inches Weight: _________ lbs**

**72. Child Height: ____ ft ______inches Weight: _________ lbs**
